# Development of a 21-miRNA Signature Associated With the Prognosis of Patients With Bladder Cancer

**DOI:** 10.3389/fonc.2019.00729

**Published:** 2019-08-07

**Authors:** Xiao-Hong Yin, Ying-Hui Jin, Yue Cao, York Wong, Hong Weng, Chao Sun, Jun-Hao Deng, Xian-Tao Zeng

**Affiliations:** ^1^Center for Evidence-Based and Translational Medicine, Zhongnan Hospital of Wuhan University, Wuhan, China; ^2^Department of Evidence-Based Medicine and Clinical Epidemiology, The Second Clinical College of Wuhan University, Wuhan, China; ^3^School and Hospital of Stomatology, Wuhan University, Wuhan, China; ^4^Department of Urology, Zhongnan Hospital of Wuhan University, Wuhan, China; ^5^Department of Orthopedic, Xinqiao Hospital of Army Medical University, Chongqing, China; ^6^Department of Orthopedic, Chinese PLA General Hospital, Beijing, China

**Keywords:** bladder cancer, miRNAs, prognostic signature, 21-miRNA signature, TCGA

## Abstract

**Objective:** To develop a prognostic signature for patients with bladder cancer (BC).

**Methods:** We identified differentially expressed miRNAs between normal bladder tissue and bladder cancer in the TCGA-BCLA dataset and evaluated prognostic values of these miRNAs. Then, a 21-miRNA signature was constructed based on the results of Cox proportional hazards regression model. Furthermore, functional enrichment analyses were conducted to explore the potential effects of the target genes of these 21 miRNAs.

**Results:** Seventy six differentially expressed miRNAs were identified, among which 21 miRNAs including hsa-let-7c, mir-143, mir-944, mir-192, mir-590, mir-490, mir-141, mir-93, mir-1-2, mir-200c, mir-133a-1, mir-1-1, mir-133b, mir-20a, mir-185, mir-19a, mir-19b-2, mir-19b-1, mir-17, mir-15a, and mir-133a-2 were demonstrated to be significantly correlated with the overall survival (OS) of bladder cancer patients using Kaplan-Meier survival analysis and Log-rank test. The results of Chi-square test and multivariable logistic regression analysis showed that the 21-miRNA signature was significantly associated with the diagnosis type and T stage of bladder cancer. Univariate and multivariable survival analyses indicated that the 21-miRNA signature was an independent factor in predicting the overall survival of patients with bladder cancer. The results of functional enrichment analysis suggested that the target genes of these 21 miRNAs were mostly enriched in critical cancer-related biological processes and pathways, and the PPI network suggested that 60 targeted genes interacted with a minimum of 30 genes were at the hub of the whole network. In addition, we performed a multivariate nomogram and decision curve analysis (DCA) to evaluate the clinical application of 21-microRNA signature.

**Conclusion:** We introduced a 21-miRNA signature which was associated the prognosis of patients of bladder cancer, and inspirational ideas for the future basic and clinical exploration.

## Introduction

Since the beginning of the twenty first century, bladder cancer (BC) has progressively become one of the most common types of cancers. BC has affected approximately 3.4 million people with 430,000 new cases each year (1). Although remarkable progresses in surgical techniques, chemotherapy, and radiotherapy have been made, the overall survival (OS) of patients with BC remains poor. What's worse, most BC patients could only be diagnosed in middle and advanced stage due to the lack of predictive indicators. As a result, the survival rate and life quality of patients remain poor owning to missing the curative surgery (2).

Micro ribonuclease acids (miRNAs), micro, non-coding RNAs of length 20–25 nt, are members of hairpin structure precursors, which are capable of interacting with the 3' non-coding region of the target mRNAs. miRNA plays an important role in post-transcriptional regulation of eukaryotic gene expression, which is crucial to cell proliferation, differentiation, migration, apoptosis, and regulation of cell cycle (3–5). At present, numerous studies have demonstrated that miRNAs could be treated as diagnostic biomarkers for multiple cancers (6, 7), and researchers come to realize the importance of better understanding of the pathogenesis of BC and identification of the new prognostic biomarkers for BC (8). Therefore, identification of prognostic miRNA biomarkers may be conducive to the diagnosis and treatment of BC (9, 10), which will contribute to the enhancement of the quality of life and survival rate of the BC patients. Thus, we analyzed the TCGA-BCLA miRNA sequencing data, evaluated the prognostic value of the differentially expressed miRNAs, and developed a 21-miRNA based signature associated with prognosis of BC patients.

## Materials and Methods

### Data Collection

The miRNA expression profiling of 453 BC patients and the corresponding clinical information were obtained from TCGA data portal (11). BC samples were excluded according to the following exclusion criteria: (1) History of other malignancies; (2) Samples with deficient miRNAs sequence expression data; (3) Repeated miRNAs sequencing samples. Then, we included 427 samples in this study, including 408 BC tissues and 19 adjacent normal bladder tissues and the corresponding clinical data (including age, gender, race, diagnosis_subtype, tumor grade, metastasis, lymph node status, and T-stage).

### Identification of Differentially Expressed miRNAs

We identified the differentially expressed miRNAs between BC tissues and adjacent normal bladder tissues using “limma” package in R language (12). miRNAs meeting |Log_2_FC| >2 and *P* < 0.05 were considered significantly expressed.

### Signature Construction and Statistical Analysis

BC samples were classified into specific miRNA low expression group and high expression group according to the median of the miRNA expression levels. Then, we identified the miRNAs closely related to the overall survival of BC patients by Log-rank test based Kaplan-Meier survival analysis. Next, miRNAs significantly correlated with the survival of BC patients were included in a multivariable Cox proportional hazards regression model on the OS of BC patients, and the risk scores of these miRNAs for each patient were calculated based on the coefficients of each miRNA in the Cox proportional hazards regression model. Thus, BC patients were categorized into “low-risk” and “high-risk” groups based on the optimal cut-off derived from time-dependent receiver operating characteristic (ROC) analysis and the ROC curve was generated by R packages “ggplot2” and “survivalROC” (13). Subsequently, the correlations between the risk score and clinical features of BC patients were analyzed by using Chi-square and multivariable logistic regression analysis. Moreover, Kaplan-Meier survival analysis, univariate and multivariable Cox proportional hazards regression analysis were performed to evaluate the survival of BC patients in low-risk group and high-risk group using the R packages “survival.” For the Cox proportional hazards regression model, we included risk score, age, diagnosis_subtype, grade, M-stage, T-stage, N-stage, gender, and race into the model, among which diagnosis_subtype, gender, and race were treated as categorized variable, and the remaining variables were treated as continuous variable. All statistical analyses were performed using the R 3.4.2 software, *P* < 0.05 was considered statistically significant (14).

### Target Gene Prediction and Function Enrichment Analysis

Target genes of prognostic miRNAs were predicted by miRDB (http://www.mirdb.org/miRDB/index.html), miRTarBase (http://mirtarbase.mbc.nctu.edu.tw/php/index.php) and TargetScan (http://www.targetscan.org/) online analysis tools. Then, overlapping genes were analyzed by the web-based online bioinformatics tool, which provided a comprehensive set of functional annotation tools to understand the biological mechanisms associated with large chains of genes or proteins. Gene Ontology (GO) enrichment analysis and Kyoto Encyclopedia of Genes and Genomes (KEGG) pathway analysis of the target genes were conducted using the database for annotation, visualization and integrated discovery (DAVID) (https://david.ncifcrf.gov/) (version 6.8), Moreover, in order to investigate the interactions between target genes, we performed the protein-protein interaction (PPI) network analysis of target genes using String Database (15).

### Clinical Application of the 21-miRNA Signature

Nomogram can be used to diagnose or predict the incidence or progress of diseases with multiple indicators (16). To clarify the clinical application ability of the 21-miRNA signature, we built a 21-miRNA based nomogram estimating the 3 and 5 year OS of BC patients, which included the age, diagnosis subtype, grade, M stage, N stage, T stage, race, and the risk score of each BC patient into a multivariate survival model. The above variables were incorporated into the multivariate survival model. Meanwhile, the prognostication value of the nomogram was verified internally using 1,000 bootstrap samples, R package “rms” was applied to draw the nomogram and to perform internal validation. Subsequently, we performed decision curve analysis (DCA) (17) to verify the clinical role of the nomogram for the 21-miRNA signature.

## Results

### Identification of Differentially Expressed miRNAs Between BC and Normal Bladder Tissue

We obtained 427 samples in this study, including 408 BC tissues and 19 adjacent normal bladder tissues, and the clinical characteristics of BC patients including age, gender, race, diagnosis_subtype, tumor grade, metastasis, lymph node status and T-stage. According to the cut-off criteria (|log_2_FC| >2 and *P* < 0.05), 76 differentially expressed miRNAs were identified between BC tissues and adjacent normal bladder tissues (Supplementary Table 1).

### Construction of the Prognostic miRNA Signature

**Figure 1:**
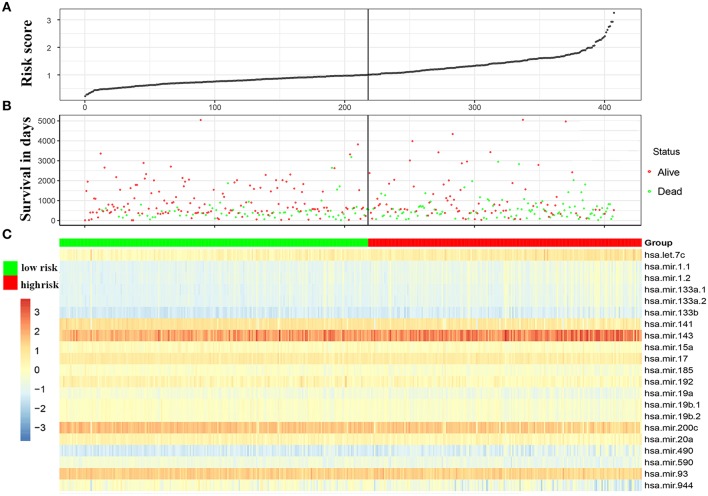
Characteristics of the 21-miRNA signature based signature. **(A)** The distribution of the 21-miRNA signature risk score for each patient. **(B)** The overall survivals of patients with bladder cancer, and their survival status. **(C)** Centered and scaled expression level of the 21 miRNAs in the signature in low-risk group and high-risk group, with cold color representing low expression and warm color representing high expression.

**Figure 2:**
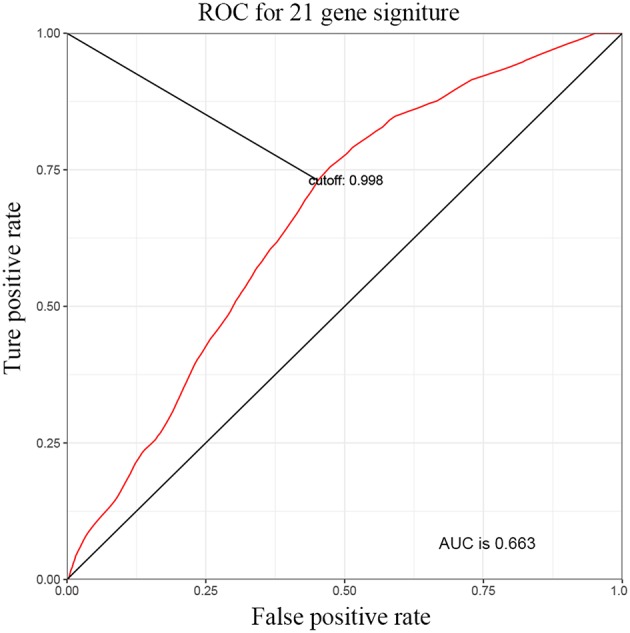
Time-dependent ROC curve for the 21-miRNA signature. Intersection of black line with ROC curve corresponds to the risk score of 0.998 which was used as cut-off for the 21-miRNA to stratify patients into low or high-risk group.

After Log-rank test based survival analysis on the expression of each miRNA and the OS of BC patients, 21 miRNAs, including are hsa-let-7c, hsa-mir-143, hsa-mir-944, hsa-mir-192, hsa-mir-590, hsa-mir-490, hsa-mir-141, hsa-mir-93, hsa-mir-1-2, hsa-mir-200c, hsa-mir-133a-1, hsa-mir-1-1, hsa-mir-133b, hsa-mir-20a, hsa-mir-185, hsa-mir-19a, hsa-mir-19b-2, hsa-mir-19b-1, hsa-mir-17, hsa-mir-15a, and hsa-mir-133a-2 were demonstrated to be significantly correlated with the OS of BC (Table 1). The 21 miRNAs were used to create a signature (named 21-miRNA signature) by calculating a risk score for each patient based on the Cox proportional hazards regression model (Figure 1 and Supplementary Table 2). According to the cutoff (0.998) of risk score, 218 BC patients were assigned to high-risk group and 190 patients were assigned to low-risk group (Figure 2).

**Table 1:** Univariate Cox proportional hazards regression analysis of 21-miRNA signature for bladder cancer.

**miRNAs**	**HR**	**LCI**	**UCI**	***P-*value**
hsa-let-7c	1.210	1.097	1.335	<0.001
hsa-mir-143	1.199	1.076	1.335	0.001
hsa-mir-944	0.920	0.864	0.981	0.010
hsa-mir-192	0.859	0.764	0.966	0.011
hsa-mir-590	0.826	0.710	0.962	0.014
hsa-mir-490	1.095	1.018	1.177	0.015
hsa-mir-141	0.909	0.842	0.982	0.016
hsa-mir-93	0.856	0.754	0.972	0.017
hsa-mir-1-2	1.109	1.018	1.208	0.018
hsa-mir-200c	0.913	0.844	0.986	0.021
hsa-mir-133a-1	1.106	1.013	1.207	0.025
hsa-mir-1-1	1.103	1.012	1.201	0.025
hsa-mir-133b	1.113	1.013	1.223	0.025
hsa-mir-20a	0.868	0.766	0.984	0.027
hsa-mir-185	0.820	0.688	0.978	0.027
hsa-mir-19a	0.863	0.756	0.985	0.029
hsa-mir-19b-2	0.847	0.724	0.990	0.037
hsa-mir-19b-1	0.847	0.724	0.991	0.038
hsa-mir-17	0.857	0.738	0.994	0.042
hsa-mir-15a	0.843	0.712	0.999	0.048
hsa-mir-133a-2	1.094	1.000	1.196	0.049

*HR, hazard ratio; LCI, lower 95% confidence interval; UCI, upper 95% confidence interval*.

### Patients in 21-miRNA Low-Risk Group Were Associated With Better Clinicopathological Features

The results of Chi-square analysis suggested that the patients in low-risk group had better clinicopathological features, including diagnosis_subtype (χ^2^ = 13.999, *P* < 0.001), tumor grade (χ^2^ = 8.009, *P* = 0.004), lymph node status (χ^2^ = 11.424, *P* = 0.009), T-stage (χ^2^ = 26.581, *P* < 0.001) and race (χ^2^ = 10.611, *P* = 0.004), compared with those in high-risk group. Based on the results of Chi-square test, diagnosis_subtype (categorized variable), tumor grade (continuous variable), lymph node status (continuous), T-stage (continuous variable), and race (categorized variable) were included into a logistic regression model, and the results confirmed that diagnostic subtypes (*OR* = 0.553, *P* = 0.024) and T-stage (*OR* = 0.874, *P* = 0.041) of patients in the high-risk group were significantly inferior to those in the low-risk group (Table 2).

**Table 2:** Correlation between the 21-miRNA signature and patients' clinicopathological features.

**Variables**	**No. of patients**	**Low *n* (%)**	**High *n* (%)**	**Univariate analysis**		^*^Multivariable analysis			
				**Chi-square**	***P*-value**	**OR**	**LCI**	**UCI**	***P*-value**
**AGE (YEARS)**									
<70	214	122 (57.01)	92 (42.99)	2.023	0.154	/	/	/	/
≧70	194	96 (49.48)	98 (50.51)						
**DIAGNOSIS_SUBTYPE**									
Non-papillary	273	129 (47.26)	144 (52.75)	13.993	<0.001	Reference			
Papillary	130	88 (67.69)	42 (32.31)			0.553	0.330	0.926	0.024
NA	5								
**TUMOR GRADE**									
Low Grade	21	18 (85.71)	3 (14.28)	8.009	0.004	0.282	0.051	1.554	0.146
High Grade	384	198 (51.56)	186 (48.44)						
NA	3								
**METASTASIS**									
M0	195	114 (58.46)	81 (41.54)	0.287	0.591	/	/	/	/
M1	11	5 (45.45)	6 (54.54)						
NA	202								
**LYMPH NODE STATUS**									
N0	237	139 (58.65)	98 (41.35)	11.424	0.009	0.770	0.587	1.012	0.061
N1	46	20 (43.48)	26 (56.52)						
N2	76	29 (38.16)	47 (61.84)						
N3	7	4 (57.14)	3 (42.85)						
NA	42								
**T-STAGE**									
T0–T1	4	3 (75.00)	1 (25.00)	26.581	<0.001	0.874	0.768	0.995	0.041
T2	120	85 (70.83)	35 (29.17)						
T3	193	80 (41.45)	113 (58.54)						
T4	58	29 (50.00)	29 (50.00)						
NA	33								
**GENDER**									
Male	301	161 (53.49)	140 (46.51)	7.585	1.00	NA	NA	NA	NA
Female	107	57 (53.27)	50 (46.72)						
**RACE**									
White	323	163 (50.46)	160 (49.53)	10.611	0.004	Reference			
Asian	44	32 (72.72)	12 (27.27)			1.034	0.405	2.735	0.945
African	23	8 (34.78)	15 (65.22)			0.476	0.170	1.231	0.136
NA	18	8 (34.78)	15 (65.22)						

* *Adjusting for Age, diagnosis_subtype, tumor grade, metastasis, lymph node status, T-stage, gender and race, respectively. Diagnosis_subtype non-papillary, and race white were set reference for variable diagnosis_subtype and race. OR, odds ratio; LCI, lower 95% confidence interval; UCI, upper 95% confidence interval; NA, not available*.

### Patients in the 21-miRNA Signature Low-Risk Group Had Better OS Compared With Those in the 21-miRNA Signature High-Risk Group

**Figure 3:**
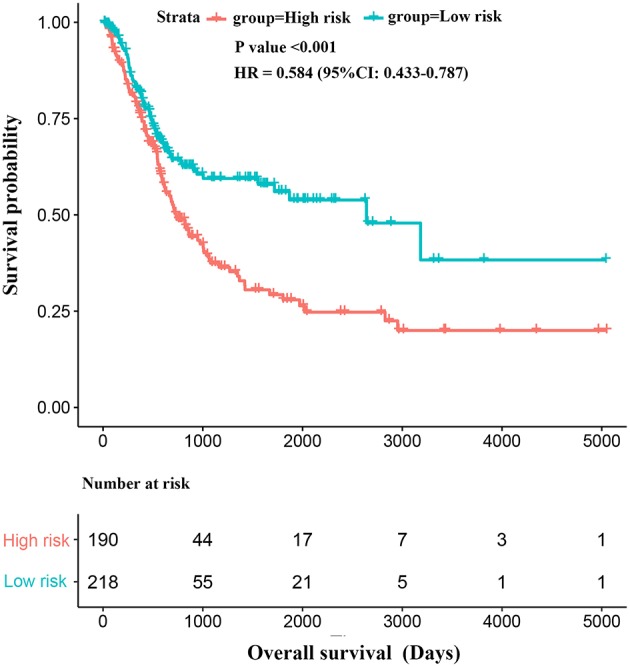
Kaplan-Meier survival analysis of overall survival of patients with bladder cancer.

In the 21-miRNA signature low-risk group, a total of 145 BC patients received transurethral resection of bladder tumor (TURBT), 45 patients received postoperative drug treatment, 8 patients received radiation therapy and 3 patients received targeted therapy. In the 21-miRNA signature high-risk group, a total of 157 patients received TURBT, 54 patients received postoperative drug treatments, 12 patients received radiation therapy, and 1 patient received targeted therapy. There were no significant differences between the two groups regarding these treatments (TURBT, postoperative drug treatment, radiation therapy, targeted therapy). Thus, Kaplan-Meier survival analysis and Log-rank statistical test were used to evaluate the difference of the survival of BC patients between the 21-miRNA signature high-risk and low-risk groups. The results showed that BC patients in the 21-miRNA signature high-risk group had shorter OS than patients in the 21-miRNA signature low-risk group (HR = 0.584, 95% CI = 0.433–0.787, *P* < 0.001; Figure 3), indicating that the 21-miRNA signature might be a potentially prognostic factor of BC. Meanwhile, the result of multivariable Cox proportional hazards regression analysis suggested that the 21-miRNA signature was an independent prognostic factor for BC patients (HR = 1.965, 95% CI = 1.151–3.354, *P* = 0.013). Moreover, the C-index for the multivariable Cox proportional hazards regression model was 0.669 (se = 0.042) (Table 3).

**Table 3:** Univariate and multivariable Cox proportional regression analysis of bladder cancer patients.

**Variables**	**Univariate analysis**		^*^Multivariable analysis	
	**HR (95% CI)**	***P*-value**	**HR (95% CI)**	***P*-value**
Risk score	2.107 (1.611~2.757)	<0.001	1.952 (1.143~3.334)	0.014
Age	1.033 (1.017~1.049)	<0.001	1.021 (0.991~1.052)	0.171
Diagnosis_subtype (Papillary)	0.677 (0.477~0.962)	0.029	1.059 (0.547~2.049)	0.864
Grade	2.913 (0.720~11.770)	0.134	1.467 (0.156~13.781)	0.738
M-stage	3.368 (1.608~7.052)	0.001	1.028 (0.307~3.432)	0.964
N-stage	1.578 (1.342~1.856)	<0.001	1.429 (1.001~2.039)	0.049
T-stage	1.219 (1.125~1.321)	<0.001	1.051 (0.899~1.229)	0.529
Gender (Female)	1.152 (0.834~1.591)	0.390	1.412 (0.724~2.75)	0.311
Race (Asian)	0.619 (0.315~1.218)	0.165	1.190 (0.402~3.519)	0.753
Race (Black or American African)	1.250 (0.709~2.204)	0.440	2.099 (0.799~5.516)	0.132

* *Adjusting for risk score, age, diagnosis_subtype, grade, M-stage, T-stage, N-stage, gender and race, respectively. Diagnosis_subtype non-papillary, and race white were set reference for variable diagnosis_subtype and race. HR, hazard ratio; CI, confidence interval*.

### Target Gene Prediction and Functional Enrichment Analysis

**Figure 4:**
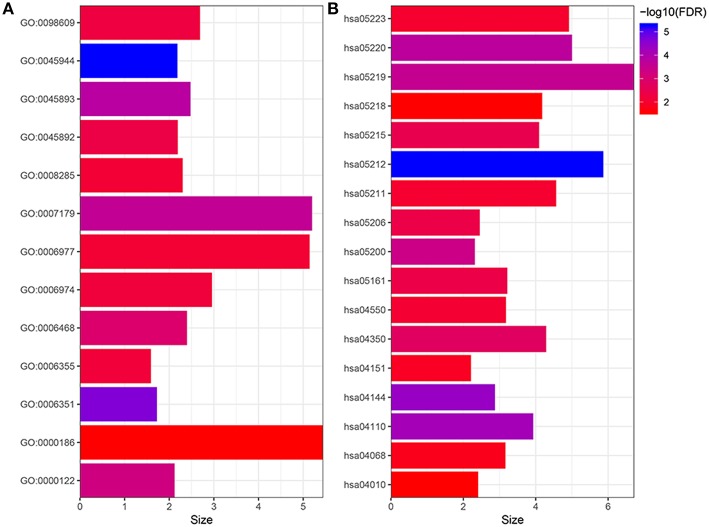
Gene ontology **(A)** and KEGG enrichment **(B)** analysis of the targets. GO:0045944, positive regulation of transcription from RNA polymerase II promoter; GO:0006351, transcription, DNA-templated; GO:0045893, positive regulation of transcription, DNA-templated; GO:0007179, transforming growth factor beta receptor signaling pathway; GO:0000122, negative regulation of transcription from RNA polymerase II promoter; GO:0006468, protein phosphorylation; GO:0045892, negative regulation of transcription, DNA-templated; GO:0098609, cell-cell adhesion; GO:0006974, cellular response to DNA damage stimulus; GO:0006355, regulation of transcription, DNA-templated; GO:0008285, negative regulation of cell proliferation; GO:0006977, DNA damage response, signal transduction by p53 class mediator resulting in cell cycle arrest GO:0000186, activation of MAPKK activity; hsa05212, Pancreatic cancer; hsa04144, Endocytosis; hsa04110, Cell cycle; hsa05220, Chronic myeloid leukemia; hsa05219, Bladder cancer; hsa05200, Pathways in cancer; hsa04350, TGF-beta signaling pathway; hsa05215, Prostate cancer; hsa05206, MicroRNAs in cancer; hsa05161, Hepatitis B; hsa04550, Signaling pathways regulating pluripotency of stem cells; hsa05211, Renal cell carcinoma; hsa05223, Non-small cell lung cancer; hsa04151, PI3K-Akt signaling pathway; hsa04068, FoxO signaling pathway; hsa05218, Melanoma; hsa04010, MAPK signaling pathway.

**Figure 5:**
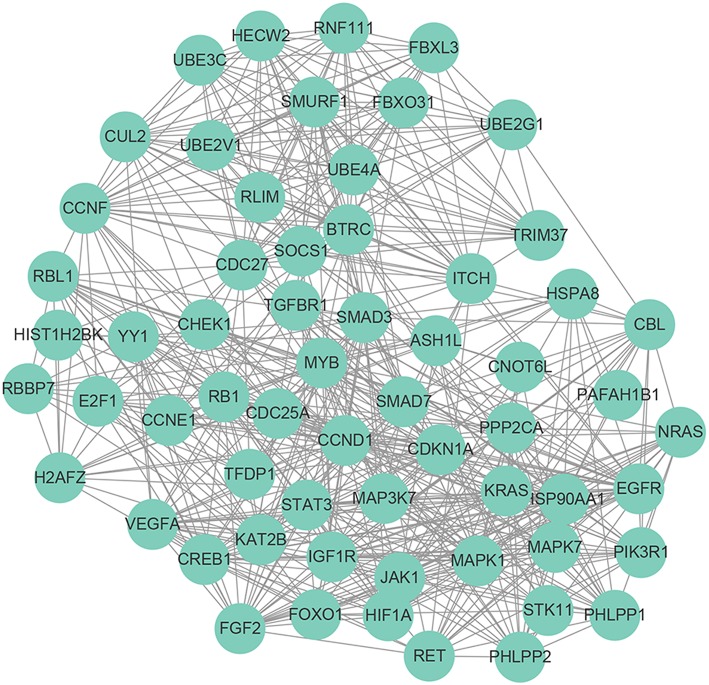
Protein-protein interaction network of the 60 targeted genes interacted with no <30 other genes.

The target genes of 21 miRNAs were predicted by using miRDB, miRTarBase, and TargetScan. As a result, 884 target genes were obtained. In order to elucidate the biological functions of the target genes, we performed the GO and KEGG signaling pathway enrichment analysis. The results of GO enrichment analysis suggested that the target genes were mainly enriched in GO terms including positive regulation of transcription from RNA polymerase II promoter, DNA-templated of transcription, positive regulation of transcription, transforming growth factor β receptor signaling pathway, negative regulation of transcription from RNA polymerase II promoter, protein phosphorylation, negative regulation of transcription, cell-cell adhesion, cellular response to DNA damage stimulus, regulation of transcription, negative regulation of cell proliferation, DNA damage response (signal transduction by p53 class mediator resulting in cell cycle arrest) and activation of MAPKK activity (Figure 4A). The results of KEGG pathway enrichment analysis suggested that these target genes were significantly (FDR <0.05) enriched in some well-known cancer related pathways, including pancreatic cancer signaling pathway, endocytosis signaling pathway, cell cycle signaling pathway, chronic myeloid leukemia signaling pathway, BC signaling pathway, pathways in cancer signaling pathway, TGF-beta signaling pathway, prostate cancer signaling pathway, MicroRNAs in cancer signaling pathway, hepatitis B signaling pathway, signaling pathways regulating pluripotency of stem cells, renal cell carcinoma signaling pathway, non-small cell lung cancer signaling pathway, PI3K-Akt signaling pathway, FoxO signaling pathway, melanoma signaling pathway and MAPK signaling pathway (Figure 4B). Finally, we conducted a PPI network analysis for the 884 target genes, as shown in Figure 5, 60 targeted genes interacted with no <30 other genes were at the hub of the whole network, indicating that these target genes played an important role in BC (Figure 5).

### Clinical Application of the 21-miRNA Signature

The 21-miRNA signature nomogram is significantly superior to the default strategies of treating all or no patient, across the threshold probabilities ranging from 31 to 82%.

**Figure 6:**
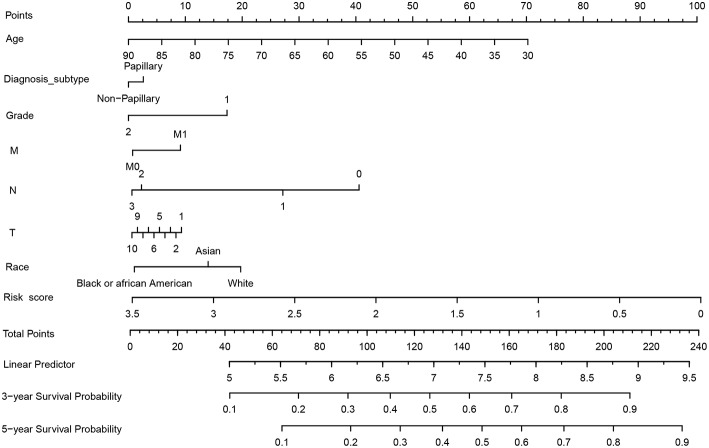
Construction of the 21-miRNA signature based nomogram and its clinical use.

**Figure 7:**
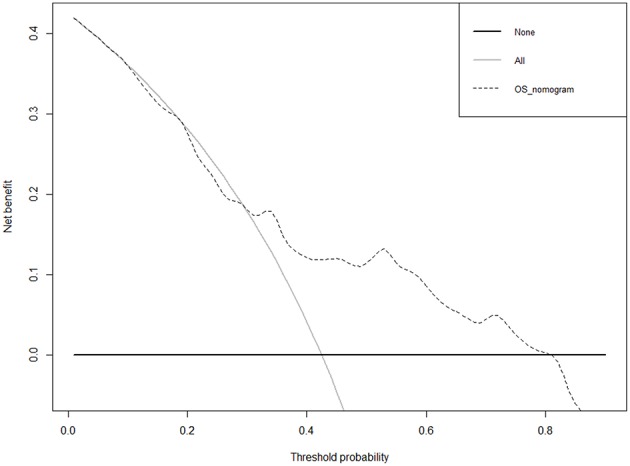
The result of decision curve analysis of the 21-miRNA signature.

As shown in Figure 6, we constructed a prognostic nomogram which included age, diagnosis subtype, grade, M stage, N stage, T stage, race, and the risk score to predict the 3 and 5 year OS of patients with BC. The result of internal validation is shown in Supplementary Table 3, and the internally validated Harrell's C-index was 0.668, suggesting that the 21-miRNA signature based nomogram showed acceptable performance in clinical settings. The total points obtained by summing the points of each variable could be used to estimate the 3 and 5 year OS rates of each patient (18). DCA, a widely accepted approach for evaluating alternative diagnostic and prognostic methods, could determine a range of threshold probabilities for a prediction model (19). The 21-miRNA signature based nomogram significantly outperformed the default strategies of treating all or none with the threshold probabilities ranging from 31 to 82% (Figure 7).

## Discussion

Gene expression signatures or miRNA-based profiling methods have been successfully used in the prevention of various tumors. In the present study, we found that the 21-miRNA signature was associated with the OS of BC patients. BC patients in the 21-miRNA signature high-risk group had shorter OS than those in the 21-miRNA signature low-risk group, and the clinicopathological features of BC patients including diagnosis_subtype, and T-stage were also demonstrated to be worse in the 21-miRNA signature high-risk group than those in the 21-miRNA signature low-risk group. Subsequently, we performed GO and KEGG pathway functional enrichment analyses to explore the potential effects and functions of these 21 miRNAs on carcinogenesis. The target genes of the 21 miRNAs were mostly enriched in critical cancer-related biological processes and pathways, such as cell proliferation, cell differentiation, cell transformation, cell cycle, endocytosis, and apoptosis. The results suggested that these miRNAs played an important role in the occurrence, development and prognosis of BC.

Through literature review of the 21 miRNAs, we found these miRNAs were mainly related to proliferation, invasion, metastasis differentiation, epithelial-to-mesenchymal transition (EMT) and angiogenesis of tumor. hsa-Let-7c might be the target of SOX4, MYC, and CCND1, and might play significant roles in colorectal cancer progression via affecting the cell cycle-related pathways and serve as crucial regulators in the p53 signaling pathway (20). High level of miR-143 could promote tumor metastasis through FNDC3B *in vitro* and *in vivo* (21). But in contrast, Xu et al. (22) suggested that miR-143 might play a role in prostate cancer cell proliferation, migration and chemosensitivity via suppressing KRAS and MAPK pathway. He et al. (23) demonstrated that miR-944 might function as an oncogene to regulate the chemoresistance of breast cancer via gain and loss function experiments *in vitro*. miR-192 (24), miR-19a (25), miR-19b, and miR-17 (26) might have enhanced the chemoresistance and invasiveness of cancer cells. miR-590 might have functioned as an oncogene by targeting nephroblastoma to induce G401 cell proliferation (27). The overexpression of miR-490-3p led to an increase in cell proliferation, migration, and invasion abilities and contributed to EMT (28). However, Zheng et al. (29) revealed that miR-490-3p suppressed cancer cell proliferation, induced apoptosis and inhibited cell invasiveness via repressing the initiation of EMT. The transcription of miR-141 and miR-200c might be directly suppressed by ZEB1, which could strongly activate epithelial differentiation of pancreatic, colorectal and breast cancer cells (30, 31).The ectopic expression of miR-93 might promote cell proliferation, migration, invasion, EMT and formation of tumor while it inhibited cell apoptosis and G1 cell cycle arrest (32). Yamasaki et al. reported that prothymosin-α (PTMA) and purine nucleoside phosphorylase (PNP) were directly regulated by miR-1-1, miR-1-2, miR-133a-1, and miR-133a-2. Silencing of these genes significantly inhibited cell proliferation, invasion and apoptosis in BC cells (33, 34). Jin et al. (35) reported that miR-15a inhibited the components of TGF-β signaling pathways in LNCaP cell line, which might be related to the progression and metastasis of prostate cancer. miR-133b and miR-20a could regulate cancer growth and chemoresistance (36, 37). Furthermore, Luengo-Gil et al. (38) indicated that miR-20a played a role in angiogenesis of BC. The miR-185 were found to be interacted with FOXD2-AS1 (39) and ITGB5 (40) might modulate proliferation, migration and invasion of cancer cells. In contrast, Zhao et al. (41) reported that miR-185 expression might suppress tumor growth by affecting KLF7 in a tumor xenograft model.

The reasons for the inconsistent results of all the above studies can be expounded as follows. First, the individual differences of different studies and small sample size might lead to the inconsistent results. Second, the regulation differences of tumors caused by mutation of genes might also lead to the inconsistency of the results (42). The third possibility may be the dysregulation caused by changes of target genes for tumor suppressors (43). The last possibility may be the functional diversity of tumor suppressor genes in different pathways of different tumors (44, 45). Therefore, we believe that the occurrence and development of BC are originally a complex process of multi-gene, multi-molecule network structure that encompasses long-term interaction with multi-steps and multi-stages. Although there are still many unknown issues in the overall regulatory mechanisms of tumors, some important discoveries have been found by virtue of the regulation of miRNAs which can interfere with the occurrence, development, clinical treatment, and prognosis of tumors.

In statistics, bootstrap method is a uniform sampling that is put back from a given training set, that is, whenever a sample is selected, it may be re-selected and added to the training set again (46). Bootstrap method provides a good idea for solving small-scale subsample test evaluation problems and is asymptotically more accurate than the standard intervals obtained using sample variance and assumptions of normality. In the present study, owning to the lack of external validation cohort, we could not validate the prognostication performance of the 21-miRNA signature by using external bladder cancer miRNA expression profile. Thus, we applied bootstrapping with 1,000 resample to internally validate the performance of the 21-miRNA signature, and the C-index for the internal validation is 0.668, which indicated an acceptable performance of the 21-miRNA signature in clinical settings.

To translate the conclusions of the present into clinical applications, we built a nomogram containing the 21-miRNA signature and other clinical features of BC patients. Users could detect the expression levels of these miRNAs and calculate the risk score of each BC patients based on the expression levels of these 21-miRNAs and their corresponding coefficients in the Cox proportional hazards model (Figure 6), then BC patients could be stratified into high risk group and low risk group based on the 21-miRNA signature and physicians could estimate the 3 and 5 year survival probabilities of BC patients. Meanwhile, the result of DCA suggested that the 21-miRNA signature containing nomogram showed better prediction ability across the threshold probabilities ranging from 31 to 82% (Figure 7).

Nevertheless, the most critical limitation of the present study is that the conclusions of the present study derived from retrospective analysis of public data and the lack of external validation of the model using *in vivo, in vitro* and prospective studies. Thus, we should keep cautious when we translate the 21-miRNA signature into clinical practice. Further large-scale and multi-center *in vivo, in vitro* and prospective clinical trials are needed in the future to confirm our new findings.

In conclusion, we introduced a 21-miRNA signature associated with the prognosis of BC patients, and it might be used as a prognostic marker in BC.

## Author Contributions

X-HY and X-TZ designed the study. X-HY, Y-HJ, and YC collected and processed the data. X-HY and HW conducted the statistical analysis. X-HY, YW, HW, CS, and J-HD wrote and revised the manuscript. X-TZ reviewed the manuscript.

### Conflict of Interest Statement

The authors declare that the research was conducted in the absence of any commercial or financial relationships that could be construed as a potential conflict of interest.
