# Supplementary material for: Development of a 21-miRNA Signature Associated With the Prognosis of Patients With Bladder Cancer
Source: Front Oncol. 2019 Aug 7;9:729. doi: 10.3389/fonc.2019.00729 (PMC6692470; doi:10.3389/fonc.2019.00729)
Supplement: Supplementary file 1 [file Table_1.DOCX]

**Supplementary table 1** 76 differentially expressed miRNAs were identified between bladder cancer tissues and adjacent normal bladder tissues.

| **miRNA** | **\|Log2FC\|** | **Ave Expr** | **adj. *P*. Value** |
| --- | --- | --- | --- |
| hsa-mir-1-2 | 3.246 | 3.738 | <0.001 |
| hsa-mir-1-1 | 3.232 | 3.654 | <0.001 |
| hsa-mir-133a-1 | 3.105 | 3.315 | <0.001 |
| hsa-mir-133a-2 | 3.013 | 3.191 | <0.001 |
| hsa-mir-133b | 2.975 | 1.837 | <0.001 |
| hsa-mir-490 | 2.956 | 2.153 | <0.001 |
| hsa-mir-1247 | 2.791 | 4.302 | <0.001 |
| hsa-mir-143 | 2.581 | 16.512 | <0.001 |
| hsa-let-7c | 2.079 | 9.201 | <0.001 |
| hsa-mir-383 | 2.039 | 1.059 | <0.001 |
| hsa-mir-516a-1 | 2.008 | 2.137 | 0.003 |
| hsa-mir-331 | 2.021 | 4.611 | <0.001 |
| hsa-mir-629 | 2.023 | 6.498 | <0.001 |
| hsa-mir-675 | 2.050 | 5.514 | 0.008 |
| hsa-mir-92a-2 | 2.063 | 12.490 | <0.001 |
| hsa-mir-92a-1 | 2.086 | 12.592 | <0.001 |
| hsa-mir-106b | 2.097 | 9.447 | <0.001 |
| hsa-mir-516a-2 | 2.101 | 2.095 | 0.001 |
| hsa-mir-671 | 2.103 | 3.371 | <0.001 |
| hsa-mir-483 | 2.127 | 3.403 | 0.010 |
| hsa-mir-32 | 2.128 | 5.197 | <0.001 |
| hsa-mir-3613 | 2.137 | 3.951 | <0.001 |
| hsa-mir-339 | 2.152 | 5.657 | <0.001 |
| hsa-mir-193b | 2.172 | 6.890 | <0.001 |
| hsa-mir-203b | 2.205 | 3.907 | 0.004 |
| hsa-mir-185 | 2.249 | 6.418 | <0.001 |
| hsa-mir-181a-2 | 2.289 | 10.404 | <0.001 |
| hsa-mir-15a | 2.289 | 7.506 | <0.001 |
| hsa-mir-181a-1 | 2.305 | 9.496 | <0.001 |
| hsa-mir-191 | 2.307 | 9.357 | <0.001 |
| hsa-mir-324 | 2.327 | 5.747 | <0.001 |
| hsa-mir-519a-1 | 2.330 | 2.294 | 0.001 |
| hsa-mir-503 | 2.347 | 3.212 | <0.001 |
| hsa-mir-19b-2 | 2.378 | 6.355 | <0.001 |
| hsa-mir-181b-2 | 2.385 | 7.160 | <0.001 |
| hsa-mir-590 | 2.419 | 4.419 | <0.001 |
| hsa-mir-181b-1 | 2.426 | 7.205 | <0.001 |
| hsa-mir-19b-1 | 2.434 | 6.489 | <0.001 |
| hsa-mir-944 | 2.434 | 5.205 | <0.001 |
| hsa-mir-142 | 2.461 | 9.757 | <0.001 |
| hsa-mir-301a | 2.598 | 4.121 | <0.001 |
| hsa-mir-584 | 2.640 | 7.745 | <0.001 |
| hsa-mir-224 | 2.645 | 5.864 | <0.001 |
| hsa-mir-192 | 2.652 | 8.229 | <0.001 |
| hsa-mir-105-2 | 2.690 | 3.286 | 0.001 |
| hsa-mir-135b | 2.706 | 3.716 | <0.001 |
| hsa-mir-21 | 2.726 | 17.953 | <0.001 |
| hsa-mir-455 | 2.741 | 7.132 | <0.001 |
| hsa-mir-708 | 2.745 | 6.590 | <0.001 |
| hsa-mir-105-1 | 2.754 | 3.272 | 0.001 |
| hsa-mir-335 | 2.758 | 6.356 | <0.001 |
| hsa-mir-17 | 2.771 | 9.885 | <0.001 |
| hsa-mir-149 | 2.789 | 6.854 | <0.001 |
| hsa-mir-345 | 2.793 | 4.132 | <0.001 |
| hsa-mir-33a | 2.813 | 4.513 | <0.001 |
| hsa-mir-1307 | 2.857 | 10.706 | <0.001 |
| hsa-mir-19a | 2.886 | 5.055 | <0.001 |
| hsa-mir-31 | 2.975 | 5.432 | <0.001 |
| hsa-mir-934 | 2.990 | 4.685 | <0.001 |
| hsa-mir-767 | 3.001 | 3.618 | 0.001 |
| hsa-mir-425 | 3.001 | 8.385 | <0.001 |
| hsa-mir-130b | 3.030 | 5.439 | <0.001 |
| hsa-mir-93 | 3.042 | 12.805 | <0.001 |
| hsa-mir-203a | 3.100 | 13.080 | <0.001 |
| hsa-mir-20a | 3.108 | 8.561 | <0.001 |
| hsa-mir-18a | 3.197 | 4.696 | <0.001 |
| hsa-mir-200c | 3.475 | 13.325 | <0.001 |
| hsa-mir-96 | 3.511 | 4.615 | <0.001 |
| hsa-mir-182 | 3.531 | 13.912 | <0.001 |
| hsa-mir-200b | 3.585 | 9.753 | <0.001 |
| hsa-mir-429 | 3.804 | 7.577 | <0.001 |
| hsa-mir-200a | 3.846 | 9.898 | <0.001 |
| hsa-mir-205 | 4.033 | 12.171 | <0.001 |
| hsa-mir-183 | 4.185 | 12.984 | <0.001 |
| hsa-mir-141 | 4.361 | 10.556 | <0.001 |
| hsa-mir-210 | 5.582 | 8.843 | <0.001 |
