# Supplementary material for: Development of a 21-miRNA Signature Associated With the Prognosis of Patients With Bladder Cancer
Source: Front Oncol. 2019 Aug 7;9:729. doi: 10.3389/fonc.2019.00729 (PMC6692470; doi:10.3389/fonc.2019.00729)
Supplement: Supplementary file 2 [file Table_2.DOCX]

**Supplementary table 2** Multivariable Cox proportional hazards regression analysis on the overall survival of patients with bladder cancer.

|  | Coefficient | HR | LCI | UCI | P value |
| --- | --- | --- | --- | --- | --- |
| hsa-let-7c | 0.1562 | 1.1690 | 1.0253 | 1.3329 | 0.0197 |
| hsa-mir-1-1 | -0.0775 | 0.9254 | 0.5652 | 1.5150 | 0.7578 |
| hsa-mir-1-2 | 0.1566 | 1.1696 | 0.7167 | 1.9086 | 0.5307 |
| hsa-mir-133a-1 | 0.1410 | 1.1514 | 0.6901 | 1.9212 | 0.5893 |
| hsa-mir-133a-2 | -0.2298 | 0.7947 | 0.5056 | 1.2489 | 0.3190 |
| hsa-mir-133b | -0.2168 | 0.8051 | 0.5742 | 1.1290 | 0.2088 |
| hsa-mir-141 | 0.2332 | 1.2626 | 0.8880 | 1.7953 | 0.1941 |
| hsa-mir-143 | 0.2715 | 1.3119 | 0.9926 | 1.7338 | 0.0564 |
| hsa-mir-15a | 0.0744 | 1.0773 | 0.8345 | 1.3907 | 0.5678 |
| hsa-mir-17 | 0.1627 | 1.1767 | 0.6014 | 2.3024 | 0.6347 |
| hsa-mir-185 | -0.0847 | 0.9188 | 0.6753 | 1.2502 | 0.5899 |
| hsa-mir-192 | -0.1162 | 0.8903 | 0.7574 | 1.0465 | 0.1587 |
| hsa-mir-19a | -0.0581 | 0.9435 | 0.5926 | 1.5023 | 0.8065 |
| hsa-mir-19b-1 | 0.0303 | 1.0308 | 0.2573 | 4.1299 | 0.9658 |
| hsa-mir-19b-2 | -0.0991 | 0.9056 | 0.2539 | 3.2306 | 0.8786 |
| hsa-mir-200c | -0.2138 | 0.8075 | 0.5747 | 1.1346 | 0.2178 |
| hsa-mir-20a | 0.0223 | 1.0226 | 0.5599 | 1.8677 | 0.9421 |
| hsa-mir-490 | 0.0264 | 1.0268 | 0.8814 | 1.1962 | 0.7345 |
| hsa-mir-590 | -0.1371 | 0.8719 | 0.6259 | 1.2145 | 0.4175 |
| hsa-mir-93 | 0.1158 | 1.1228 | 0.8417 | 1.4978 | 0.4308 |
| hsa-mir-944 | -0.0574 | 0.9442 | 0.8692 | 1.0257 | 0.1738 |

**Abbreviations:** HR, hazards ratio; LCI, lower limit of confidence interval; UCI, upper limit of confidence interval.
