# Supplementary material for: Development of a 21-miRNA Signature Associated With the Prognosis of Patients With Bladder Cancer
Source: Front Oncol. 2019 Aug 7;9:729. doi: 10.3389/fonc.2019.00729 (PMC6692470; doi:10.3389/fonc.2019.00729)
Supplement: Supplementary file 3 [file Table_3.DOCX]

**Supplementary table 3** Internal validation of the 21-miRNA containing Cox proportional hazards regression model with 1000 bootstrap resamples.

|  | index original | training | test | optimism | index corrected | n |
| --- | --- | --- | --- | --- | --- | --- |
| **Dxy** | **0.3351** | **0.4001** | **0.2834** | **0.1167** | **0.2185** | **1000** |
| R2 | 0.1223 | 0.1682 | 0.0828 | 0.0854 | 0.0369 | 1000 |
| Intercept | 0 | 0 | 2.5835 | -2.5835 | 2.5835 | 1000 |
| Slope | 1 | 1 | 0.6495 | 0.3505 | 0.6495 | 1000 |
| D | 0.0189 | 0.0276 | 0.0122 | 0.0154 | 0.0036 | 1000 |
| U | -0.0020 | -0.0020 | -0.0082 | 0.0062 | -0.0082 | 1000 |
| Q | 0.0209 | 0.0296 | 0.0204 | 0.0091 | 0.0118 | 1000 |
| g | 0.8047 | 1.1663 | 0.6948 | 0.4715 | 0.3332 | 1000 |

***** Dxy, Somers’ Dxy; R2, Nagelkerke R2; D, the discrimination index D; U, the unreliability index U ((difference in -2 log likelihood between uncalibrated Xβ and Xβ with overall slope calibrated to test sample) / L); Q, the overall quality index (Q = D−U); g, the g-index on the log relative hazard (linear predictor) scale.

Statistics validated include the Nagelkerke R2 , Dxy, slope shrinkage, the discrimination index D [(model L.R. χ 2 - 1)/L], the unreliability index U = (difference in -2 log likelihood between uncalibrated Xβ and Xβ with overall slope calibrated to test sample) / L, and the overall quality index Q = D−U. g is the g-index on the log relative hazard (linear predictor) scale; The values corresponding to the row Dxy are equal to 2 ∗ (C − 0.5) where C is the C-index or concordance probability.
